# Supplementary figures and images for: Efficient data transmission on wireless communication through a privacy-enhanced blockchain process
Source: PeerJ Comput Sci. 2023 Apr 21;9:e1308. doi: 10.7717/peerj-cs.1308 (PMC10280508; doi:10.7717/peerj-cs.1308)

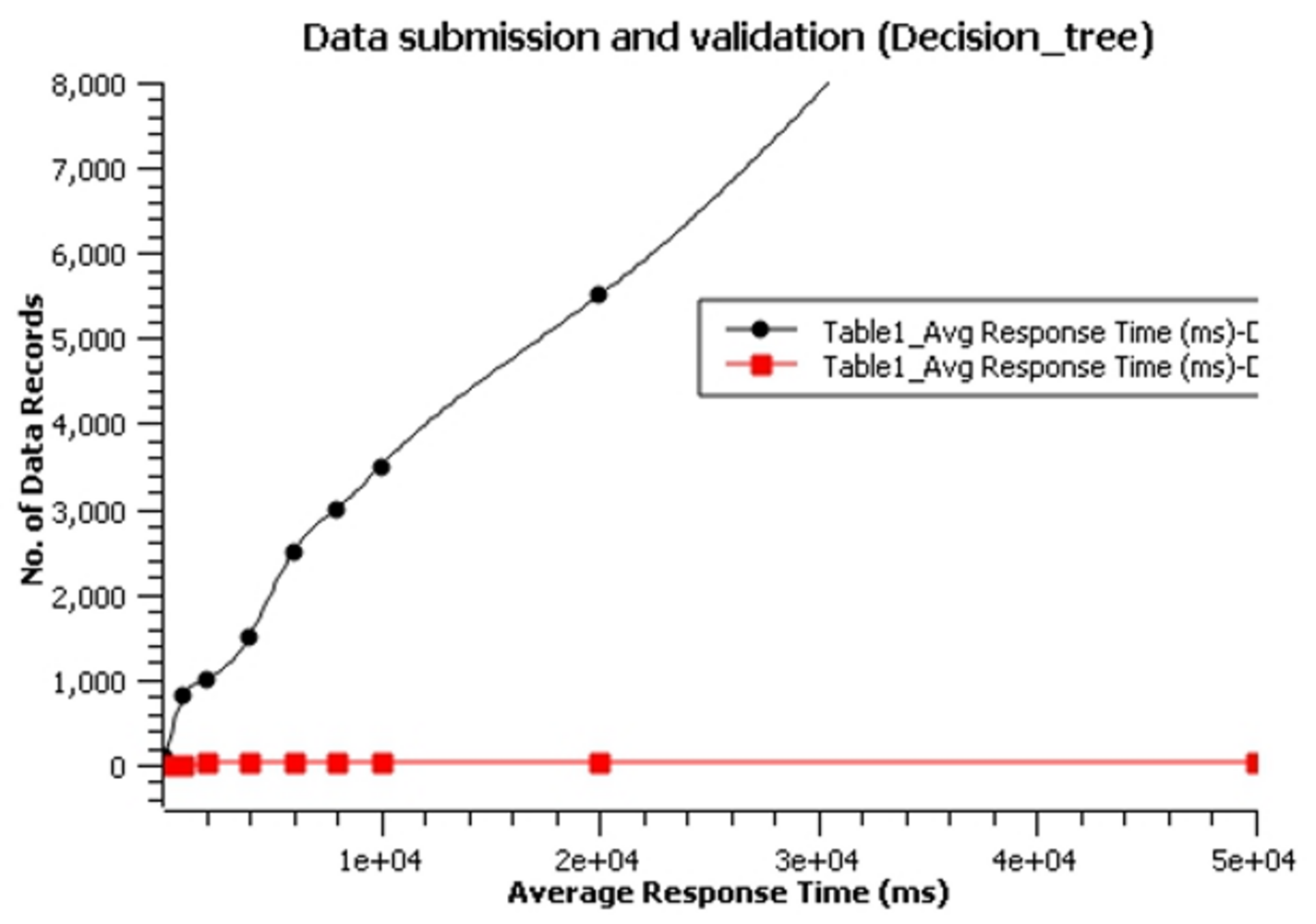

Supplement: Supplemental Information 1 [file peerj-cs-09-1308-s001.png]

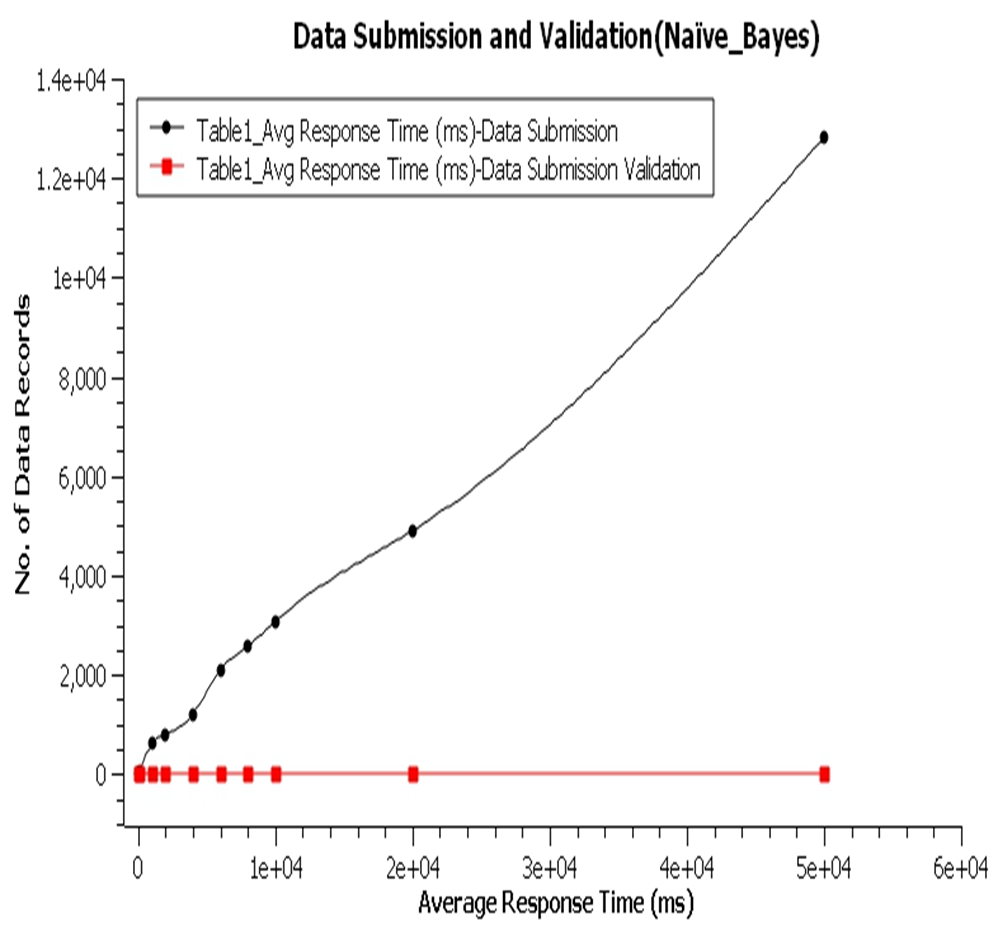

Supplement: Supplemental Information 2 [file peerj-cs-09-1308-s002.png]

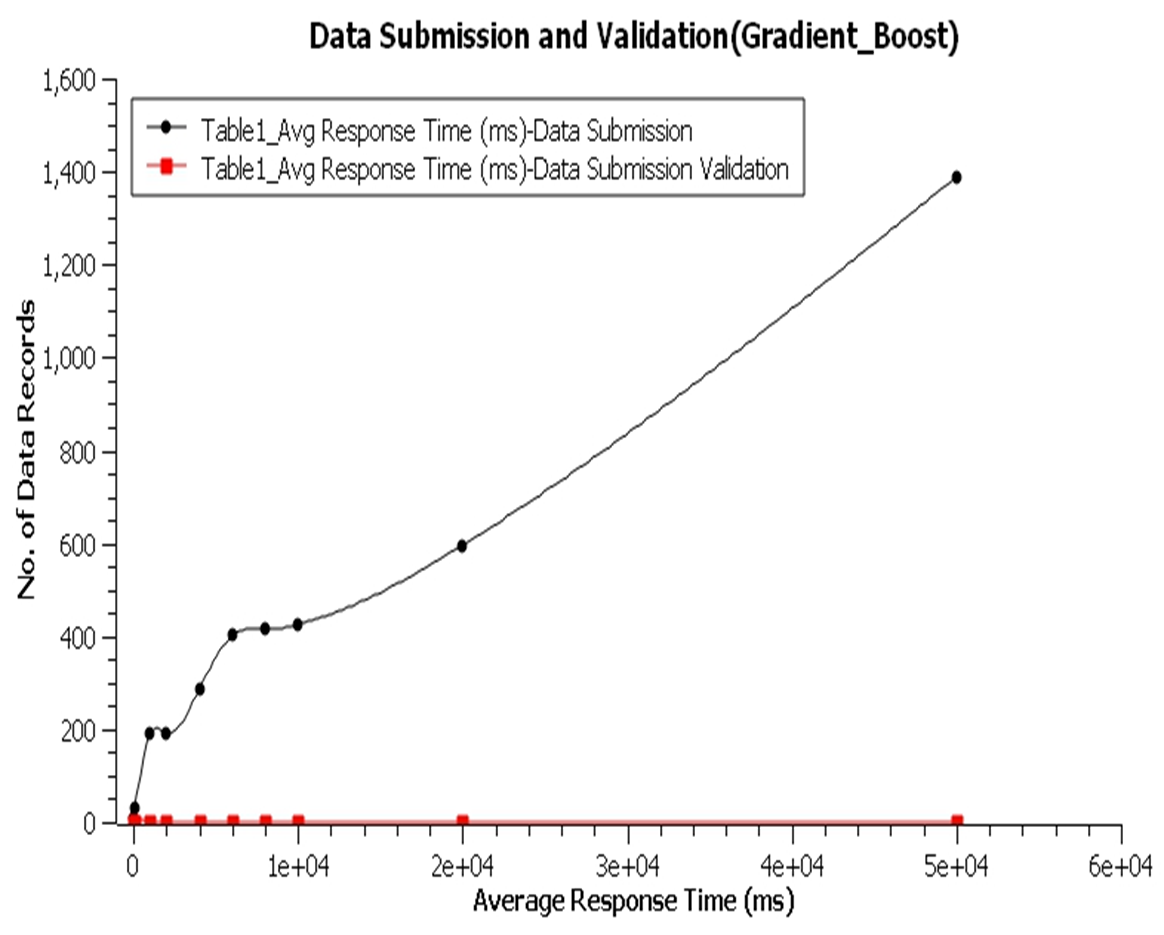

Supplement: Supplemental Information 3 [file peerj-cs-09-1308-s003.png]
