# Supplementary material for: Efficient data transmission on wireless communication through a privacy-enhanced blockchain process
Source: PeerJ Comput Sci. 2023 Apr 21;9:e1308. doi: 10.7717/peerj-cs.1308 (PMC10280508; doi:10.7717/peerj-cs.1308)
Supplement: Supplemental Information 4 [file peerj-cs-09-1308-s004.docx]

**Table 4. Analysis of average response time data submission and validation**

| **Methods** | **Decision Tree** | | **Naïve Bayes** | | **Gradient Boost** | |
| --- | --- | --- | --- | --- | --- | --- |
|  | **Data submission** | **Data validation** | **Data submission** | **Data validation** | **Data submission** | **Data validation** |
| **No. of Data records** | **Average Response Time (ms)** | **Average Response Time (ms)** | **Average Response Time (ms)** | **Average Response Time (ms)** | **Average Response Time (ms)** | **Average Response Time (ms)** |
| 10 | 20 | 0.300 | 11.031 | 0.173 | 8.969 | 0.127 |
| 100 | 100 | 4.300 | 69.040 | 3.315 | 30.960 | 0.985 |
| 1000 | 800 | 8.200 | 610.875 | 6.912 | 189.125 | 1.288 |
| 2000 | 1000 | 14.300 | 808.795 | 12.591 | 191.205 | 1.709 |
| 4000 | 1500 | 19.000 | 1213.193 | 17.167 | 286.807 | 1.833 |
| 6000 | 2500 | 21.200 | 2098.716 | 19.486 | 401.284 | 1.714 |
| 8000 | 3000 | 24.700 | 2585.062 | 22.981 | 414.938 | 1.719 |
| 10000 | 3500 | 26.800 | 3074.727 | 25.162 | 425.273 | 1.638 |
| 20000 | 5500 | 27.800 | 4904.117 | 26.286 | 595.883 | 1.514 |
| 50000 | 14200 | 29.800 | 12811.926 | 28.337 | 1388.074 | 1.463 |
